# Efficacy, safety, and cost-effectiveness analysis of Cerebrolysin in acute ischemic stroke: A rapid health technology assessment

**DOI:** 10.1097/MD.0000000000037593

**Published:** 2024-03-29

**Authors:** Miaomiao Wan, Ke Yang, Gonghao Zhang, Chunxia Yang, Yuqing Wei, Yeqian He, Xia Jiang

**Affiliations:** a Department of Clinical Pharmacy, The First Affiliated Hospital of Guangxi Medical University, Nanning, China; b College of First Clinical Medical, Guangxi Medical University, Nanning, China; c College of Pharmacy, Guangxi Medical University, Nanning, China.

**Keywords:** A Measure Tool to Assess Systematic Reviews 2, acute ischemic stroke, Cerebrolysin, Consolidated Health Economic Evaluation Reporting Standards 2022, rapid health technology assessment

## Abstract

This study conducts a rapid health technology assessment to systematically evaluate the effectiveness, safety, and cost-effectiveness of Cerebrolysin as an adjunctive therapy for acute ischemic stroke to provide evidence-based medicine for clinical decisions of Cerebrolysin. All systematic reviews/meta-analyses, pharmacoeconomic studies, and health technology assessment reports of Cerebrolysin for the treatment of acute ischemic stroke before August 17, 2023, were retrieved from PubMed, Embase, Cochrane Library, China National Knowledge Infrastructure, Wanfang, Weipu, Sinomed database and the official website of health technology assessment. According to the inclusion and exclusion criteria, 2 researchers independently carried out screening, data extraction, and quality evaluation and descriptively analyzed the results of the included studies. A total of 14 pieces of literature were incorporated, comprising 8 systematic reviews/meta-analyses and 6 pharmacoeconomic studies. In terms of effectiveness, compared to control groups, the use of Cerebrolysin as a treatment for acute ischemic stroke demonstrates certain advantages, including enhancement in total efficacy rate, neurological function, upper limb motor dysfunction, and facilitation of the recovery of activities of daily living. Especially in patients with moderate to severe acute ischemic stroke, Cerebrolysin has demonstrated the ability to enhance neurological function recovery and ameliorate disabilities. Regarding safety, adverse reactions were mild or comparable to those in the control group. The primary findings of economic studies reveal that advocating for the use of Cerebrolysin offers certain cost-effectiveness advantages. Cerebrolysin contributes to improved clinical efficacy and evaluation indexes while demonstrating favorable safety and economic benefits.

## 1. Introduction

Acute ischemic stroke (AIS) refers to the softening and necrosis of local brain tissue due to blood circulation disorders, ischemia, and hypoxia. Over the past 30 years, 80% of developing countries have witnessed a shift in disease patterns from infectious to non-communicable, with stroke emerging as one of the most prevalent debilitating conditions. Current global stroke statistics reveal that stroke remains the second-leading cause of death and the third-leading cause of mortality and disability worldwide. Ischemic stroke (IS), comprising 70% of all strokes, is associated with a high risk of long-term and recurrence occurrences.^[1,2]^ AIS is characterized by elevated disability and mortality rates, posing a significant threat to public health and increasing the economic burden on families and society.

Currently, confirmed effective treatments for early recanalization in AIS primarily encompass intravenous thrombolysis and endovascular therapy.^[3]^ The prompt reopening of obstructed vessels serves to salvage the ischemic penumbra. However, due to strict eligibility criteria, including imaging characteristics and a narrow therapeutic time window, only a minority of stroke patients qualify for intravenous thrombolysis, endovascular clot retrieval, or a combination of both therapies. Hence, there is a pressing demand for adjuvant neuroprotective therapy.^[4]^ Diverging from exogenous or indirect neuroprotection strategies primarily focused on the cerebrovascular system, such as averting thrombus formation or disassembling existing clots, neuroprotection is defined as “neuronal preservation.” The objective of employing neurotrophic drugs for neuroprotection is to intervene in the pathological biochemical cascade within the ischemic penumbra, thereby preventing or delaying neuronal cell death.^[5]^ Cerebrolysin, one of the neurotrophic drugs, consists of a mixture of low molecular weight porcine-derived peptides and free amino acids.^[6]^ Currently, Cerebrolysin is primarily used to treat cerebral ischemia and neurodegenerative diseases.^[7]^ However, despite numerous trials, the current guidelines do not strongly recommend or discourage its use.^[8–10]^

Health technology assessment (HTA) provides decision-makers with insights to facilitate the establishment of an equitable, efficient, and high-quality healthcare system.^[11]^ The process entails a comprehensive evaluation of the technical characteristics, clinical safety, effectiveness, economic impact, equity, and ethical considerations associated with healthcare technology.^[12]^ However, one drawback of HTA is its potential for being time-consuming. In contrast, the Rapid Health Technology Assessment (RHTA) entails swiftly evaluating a specific health technology’s efficacy, safety, and cost-effectiveness. Typically completed within 1 to 6 months, RHTA enables the timely provision of crucial information for informed policy decisions while encompassing all essential aspects expected from a comprehensive review.^[13]^ This study was expected to evaluate the efficacy, safety, and cost-effectiveness of adjuvant treatment of AIS with Cerebrolysin by RHTA to provide evidence-based evidence for the rational clinical use of Cerebrolysin.

## 2. Methods

### 2.1. Inclusion and exclusion criteria

#### 2.1.1. Types of research.

We included published systematic reviews (SR) or meta-analyses, pharmacoeconomic studies, and HTA reports.

#### 2.1.2. Research subjects.

The study population was patients who met the diagnostic criteria for AIS.^[14]^

#### 2.1.3. Interventions.

The treatment group consisted of Cerebrolysin monotherapy or a combination with conventional treatment. The control group received either conventional treatment, placebo combined with conventional treatment, or other neurotrophic drugs along with conventional treatment. The treatment and control groups had no limitations on drug dosage or duration.

Conventional treatment was defined as pharmacological and non-pharmacological treatments excluding neuroprotective agents, including improvement of cerebral blood circulation (intravenous thrombolysis, endovascular therapy, antiplatelet treatment, antithrombotic treatment, fibrinolysis therapy, volume expansion, etc.) and symptomatic treatments (respiration and supplemental oxygen, cardiac monitoring, temperature control, blood pressure control, plasma glucose control, and lipid control, etc.).^[9]^

#### 2.1.4. Outcome indicators.

Efficacy indicators include overall clinical efficacy rate, cure rate, neurological deficit assessment, stroke-related motor outcome measure, daily living ability assessment, hemorheology-related indicators, etc. Safety indicators include the incidence of all-cause mortality, adverse reactions (AR), adverse events (AE), serious adverse reactions (SAR), serious adverse events (SAE), disability rate, etc. Economic indicators include the cost-effectiveness ratio and the incremental cost-effectiveness ratio.

#### 2.1.5. Exclusion criteria.

The exclusion criteria were as follows: study population: literature on clinical studies not targeting IS, such as neonatal hypoxic-ischemic encephalopathy and acute hemorrhagic stroke; study measures: the combination of drugs (Cerebrolysin combined with Shuxuetong injection, etc.) and combination of non-pharmacological treatments (hyperbaric oxygen, etc.); updated literature published by the same author in different years, taking the latest; and irrelevant literature, reviews, conference abstract, literature with lack of data or inability to obtain the complete text, and animal experiments, etc.

### 2.2. Search strategy

We searched databases including PubMed, Embase, the Cochrane Library, China National Knowledge Infrastructure, Wanfang, Weipu, and SinoMed. Meanwhile, we searched for HTA in the *International Network of Agencies for Health Technology Assessment, International Society of Technology Assessment in Health Care*, and *Health Technology Assessment international* (HTAi). We were conducted using “stroke, Cerebrolysin, Pharmacy Economic, Meta-Analysis, etc.” as subject terms, with a search time frame from the date of database inception to August 17, 2023. In addition, the references of the included studies were manually searched to ensure completeness. The search strategy is shown in Table S1, Supplemental Digital Content, http://links.lww.com/MD/L981, using PubMed as an example.

### 2.3. Literature screening

After deduplication using Endnote X9, two researchers (M.W. and K.Y.) independently screened and crosschecked literature by reading the title, abstract, and full text according to the inclusion and exclusion criteria. If there were any disagreement, they would negotiate with the third researcher.

### 2.4. Literature extraction

It was independently extracted by 2 researchers (M.W. and K.Y.) in accordance with a predesigned data extraction form. Table 1 presents the essential characteristics of systematic evaluations/meta-analysis that reflect effectiveness and safety, including the first author, publication year, type of study, number of people in the study, intervention vs. control comparisons, risk of bias assessment methods, etc. The primary characteristics of pharmacoeconomic studies reflecting economy are shown in Table 2, consisting of the first author, publication year, geographical region, research perspective, intervention vs. control comparisons, research methodology, etc.

**Table 1:** Summary of included SRs/meta-analyses.

Study	Research type	Retrieval date	Study population	Intervention measure		Bias risk assessment method	No. (cases)	Total cases	Efficacy outcome index	Safety outcome index
				Comparison group	Control group					
Zhang, D 2017^[15]^	Meta-analysis	2016.7	Acute ischemic stroke	Cerebrolysin monotherapy or combination with conventional treatment	Placebo or placebo + conventional treatment	Cochrane Risk of bias tool	7	1779	(5)(6)	(8)(9)(10)
Wang, Z 2017^[16]^	Meta-analysis	1980.1–2016.5	Acute ischemic stroke	Cerebrolysin in combination with conventional treatment	Placebo + conventional treatment	Cochrane Risk of bias tool	6	1649	(2)(5)(6)	(8)(9)(10)
Bornstein, N. M 2017^[17]^	Meta-analysis	2016.12.31	Acute ischemic stroke	Cerebrolysin in combination with conventional treatment	Placebo + conventional treatment	Jadad scale	9	1879	(2)(6)	(8)(9)(10)
Guekht, A 2017^[18]^	Meta-analysis	/	Acute ischemic stroke	Cerebrolysin	Placebo	Not reported	2	448	(2)(4)	(8)(9)(10)
Tang R 2017^[19]^	Systematic review	2016.7	Acute cerebral infarction	Cerebrolysin in combination with conventional treatment	Conventional treatment or placebo + conventional treatment	Cochrane Risk of bias tool	20	3313	(1)(2)(5)	(8)(9)(10)(11)
Yu F 2018^[20]^	Meta-analysis	2017.9	Acute ischemic stroke	Cerebrolysin in combination with conventional treatment	Conventional treatment or placebo + conventional treatment or other neurotrophic drugs + conventional treatment	Cochrane Risk of bias tool	31	3203	(1)(2)(3)(5)(7)	(9)
Ziganshina, L. E 2020^[21]^	Systematic review	2019.10.24	Acute ischemic stroke	Cerebrolysin in combination with conventional treatment	Conventional treatment or placebo + conventional treatment	Cochrane Risk of bias tool	7	1601	/	(8)(9)(10)(12)
Strilciuc, S 2021^[22]^	Systematic review + meta-analysis	2021.2.28	Acute ischemic stroke	Cerebrolysin in combination with conventional treatment	Placebo + conventional treatment	Not reported	12	2274	/	(8)(9)(10)

(1) Overall clinical efficacy rate: The proportion of patients who achieved basic recovery, significant improvement, and improvement among all cases; Neurological deficit assessment: (2) The National Institutes of Health Stroke Scale (NIHSS), (3) Modified Edinburgh-Scandinavia stroke scale (MESSS); Stroke-related motor outcome measures: (4) The Action Research Arm Test (ARAT) Scale^[23]^; Daily living ability assessment^[24]^: (5) Barthel Index Scale(BI) and (6) modified Rankin Scale(mRS). (7) Hemorheology-related indicators: whole blood viscosity (high shear rate, low shear rate), plasma viscosity, and fibrinogen content. Safety indicators: (8) Mortality rate, (9) Adverse reaction (AR)/adverse event (AE), (10) Serious adverse reaction (SAR)/serious adverse event (SAE), (11) Disability rate, (12) Non-death attrition.

Other neurotrophic drugs are Citicoline, Edaravone, etc.

**Table 2:** Summary of included Pharmacoeconomic studies.

Study	Location	Research view	Economic model	Disease	Intervention measure		Economic research methods	Economic research indexes
					Comparison group	Control group		
Lin WS 2004^[25]^	China	Not reported	Not used	Acute ischemic stroke	Cerebrolysin (Bi’aoxing, Lizhusaile) + conventional treatment	Citicoline + conventional treatment	Cost-Effectiveness Analysis	Incremental cost effectiveness ratio (△C/△E)
Zhang GX 2010^[26]^	China	Not reported	Not used	Cerebral infarction	Cerebrolysin+ conventional treatment	Acanthopanax senticosus injection + conventional treatment/Edaravone + conventional treatment	Cost-Effectiveness Analysis	Incremental cost effectiveness ratio (△C/△E)
Li G 2013^[27]^	China	Not reported	Not used	Acute cerebral infarction	Cerebrolysin+ conventional treatment	Gangliosides + conventional treatment	Cost-Effectiveness Analysis	Cost effectiveness ratio (C/E)
Men P 2016^[28]^	China	Not reported	Not used	Acute ischemic stroke	Cerebrolysin + conventional treatment	Conventional treatment	Cost-Effectiveness Analysis	Incremental cost effectiveness ratio (△C/△E)
He X 2017^[29]^	China	Not reported	Not used	Acute ischemic stroke	Cerebrolysin (Ninzhexin, Shuratai, and Qu’ao) + conventional treatment	Conventional treatment	Cost-Effectiveness Analysis	Incremental cost effectiveness ratio (△C/△E)
Strilciuc, S 2023^[30]^	Romanian	A payer perspective	Not used	Acute ischemic stroke	Cerebrolysin	Placebo	Cost-Effectiveness Analysis	Incremental cost effectiveness ratio (△C/△E)

### 2.5. Quality evaluation

The methodological quality of the included SR or meta-analyses was evaluated using the A Measure Tool to Assess Systematic Reviews 2 (AMSTAR 2) scale, consisting of 16 items, with items 2, 4, 7, 9, 11, 13, and 15 considered vital items.^[31]^ The quality of the pharmacoeconomic studies was assessed using the Consolidated Health Economic Evaluation Reporting Standards (CHEERS 2022) checklist.^[32]^

### 2.6. Data consolidation and analysis

We conducted a literature review, analyzed the fundamental characteristics of the included studies, and summarized the results using a combination of descriptive analysis and tables.

## 3. Result

### 3.1. Literature search results

**Figure 1.**
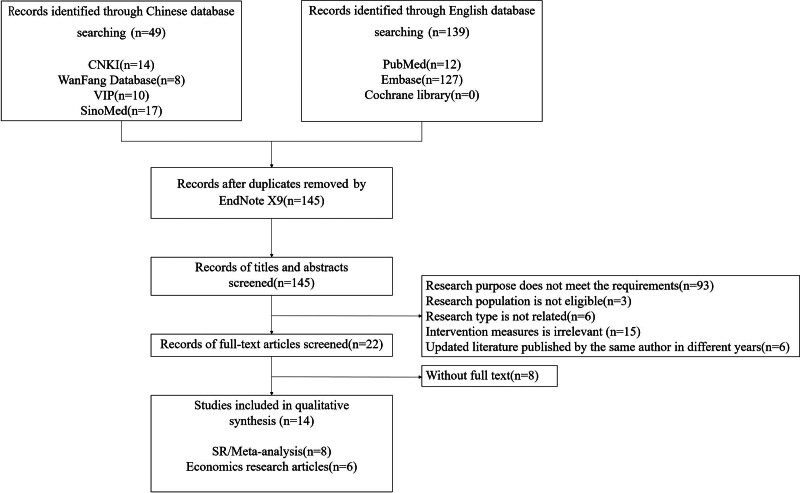
Literature screening flow chart.

In the initial retrieval, a total of 188 articles were identified. After layer-by-layer screening according to the inclusion and exclusion criteria, a final set of 14 articles was included, including 8 SR or meta-analysis^[15–22]^ and 6 pharmacoeconomic studies.^[25–30]^ No HTA report was retrieved. The literature screening process and results are provided in Figure 1.

### 3.2. Main characteristics of the included literature

The main features of the included studies are reported in Tables 1 and 2.

### 3.3. Quality evaluation of included studies

#### 3.3.1. Quality evaluation of SR/meta-analysis.

Seven SR/meta-analyses were included in the study, and the indicators of clinical effectiveness evaluation included: Overall clinical efficacy rate; Neurological deficit assessment: National Institutes of Health Stroke Scale(NIHSS) Score and Modified Edinburgh-Scandinavian Stroke Scale (MESSS) Score; Stroke-related motor outcome measures: The Action Research Arm Test (ARAT) Scale;^[23]^ Daily living ability assessment: Barthel Index Scale(BI) score and modified Rankin Scale(mRS) score;^[24]^ and Hemorheology related indicators: whole blood viscosity, plasma viscosity, and fibrinogen content. Safety indicators include disability rate, mortality rate, AE, SAE, AR, SAR etc. We utilized the AMSTAR2 scale to assess the quality of the included studies. The quality evaluation results of the SR/Meta-analysis are shown in Table 3

**Table 3:** Quality evaluation of included SR/meta-analysis.

Items	Study							
	Zhang, D 2017^[15]^	Wang, Z 2017^[16]^	Bornstein, NM 2017^[17]^	Guekht, A 2017^[18]^	Tang, R 2017^[19]^	Yu, F 2018^[20]^	Ziganshina, LE 2020^[21]^	Strilciuc, S 2021^[22]^
1. Did the research questions and inclusion criteria for the review include the components of PICO?	Y	Y	Y	Y	Y	Y	Y	Y
2. Did the report of the review contain an explicit statement that the review methods were established prior to the conduct of the review and did the report justify any significant deviations from the protocol?	N	PY	PY	N	N	N	Y	Y
3. Did the review authors explain their selection of the study designs for inclusion in the review?	N	N	N	Y	N	N	Y	N
4. Did the review authors use a comprehensive literature search strategy?	PY	N	Y	N	PY	N	Y	Y
5. Did the review authors perform study selection in duplicate?	Y	Y	Y	N	Y	Y	Y	Y
6. Did the review authors perform data extraction in duplicate?	N	Y	Y	N	N	Y	Y	Y
7. Did the review authors provide a list of excluded studies and justify the exclusions?	N	N	N	N	N	N	Y	N
8. Did the review authors describe the included studies in adequate detail?	Y	Y	Y	PY	N	PY	Y	Y
9. Did the review authors use a satisfactory technique for assessing the risk of bias (RoB) in individual studies that were included in the review?	Y	Y	Y	N	Y	Y	Y	Y
10. Did the review authors report on the sources of funding for the studies included in the review?	N	N	N	N	N	N	Y	N
11. If meta-analysis was performed did the review authors use appropriate methods for statistical combination of results?	Y	Y	Y	Y	N	N	Y	Y
12. If meta-analysis was performed, did the review authors assess the potential impact of RoB in individual studies on the results of the meta-analysis or other evidence synthesis?	N	N	Y	Y	N	N	Y	Y
13. Did the review authors account for RoB in individual studies when interpreting/discussing the results of the review?	Y	N	N	N	Y	Y	Y	Y
14. Did the review authors provide a satisfactory explanation for, and discussion of, any heterogeneity observed in the results of the review?	Y	Y	Y	Y	N	N	Y	Y
15. If they performed quantitative synthesis did the review authors carry out an adequate investigation of publication bias (small study bias) and discuss its likely impact on the results of the review?	Y	Y	Y	N	N	N	Y	N
16. Did the review authors report any potential sources of conflict of interest, including any funding they received for conducting the review?	Y	Y	N	Y	N	N	Y	Y
The number of key items not compliant	2	3	2	6	4	5	0	2
The number of non-key items not compliant	4	3	3	3	7	5	0	2
Grades	Critically low	Critically low	Critically low	Critically low	Critically low	Critically low	High	Critically low

Yes (Y): The report content adheres to the standard of a specific item on the AMSTAR2 scale; Partial Yes (PY): The report content is not comprehensive and partially adheres to the standard of a specific item on the AMSTAR2 scale; No (N): The report content does not comply with the standard of a specific item on the AMSTAR2 scale.

Following the evaluation criteria outlined in the AMSTAR 2 guidelines,^[31]^ the results indicate that, with the exception of one Cochrane systematic review assessed as “high” quality, the quality of the remaining included literature is categorized as “critically low.” The primary factors contributing to the low methodological quality of the included studies are as follows: the report of the review did not state an explicit statement that the research methods for the systematic review had been established prior to its implementation^[15,18–20]^; lack of comprehensiveness in the literature retrieval strategies^[16,18,20]^; except for a few studies,^[21]^ the review authors did not provide a list of excluded studies and justify the exclusions; there were incomplete items to assess the risk of bias^[18]^; the review authors did not describe the reason of combining the data in a meta-analysis^[19]^; the effect of the risk of bias on the results was not discussed^[16–18]^; the publication bias was not reported^[18,22]^ or the severity degree of the impact of publication bias was not discussed.^[19]^

#### 3.3.2. Quality evaluation of economic research.

Including 6 pharmacoeconomic evaluations, the efficacy indicators comprised changes between pre-and post-treatment NIHSS scores, BI index, the total effective rate assessed according to the Degree of clinic neurological function deficit scale (NDS) in Stroke Patients (1995), and so on. Economic evaluation indicators encompassed the cost-effectiveness ratio (C/E) and the incremental cost-effectiveness ratio (△C/△E), among others. The quality evaluation results of economic research are shown in Table 4.

**Table 4:** Quality evaluation of included pharmacoeconomic studies.

Items		Study					
		Lin WS 2004^[25]^	Zhang GX 2010^[26]^	Li G 2013^[27]^	Men P 2016^[28]^	He X 2017^[29]^	Strilciuc S 2023^[30]^
1. Title		PC	PC	C	C	C	C
2. Abstract		C	C	C	C	C	C
3. Background and objectives		PC	PC	PC	C	C	C
4. Health economic analysis plan		NR	NR	NR	NR	NR	NR
5. Study population		C	C	C	C	C	NR
6. Setting and location		NA	NA	NA	NA	NA	C
7. Comparators		PC	PC	PC	PC	PC	C
8. Perspective		NR	NR	NR	NR	NR	PC
9. Time horizon		PC	PC	PC	PC	PC	PC
10. Discount rate		NR	NR	NR	NR	NR	PC
11. Selection of outcomes		PC	PC	C	PC	PC	PC
12. Measurement of outcomes		C	C	C	C	C	C
13. Valuation of outcomes		C	C	NR	NR	C	C
14. Measurement and valuation of resources and costs		C	C	NR	C	C	C
15. Currency, price date, and conversion		PC	NR	NR	NR	NR	C
16. Rationale and description of model		NA	NA	NA	NA	NA	NA
17. Analytics and assumptions		C	C	C	C	C	C
18. Characterizing heterogeneity		NA	NA	NA	NA	NA	NA
19. Characterizing distributional effects		NA	NA	NA	NA	PC	NA
20. Characterizing uncertainty		C	NR	NR	C	C	C
21. Approach to engagement with patients and others affected by the study		NA	NA	NA	NA	NA	NA
22. Study parameters		NA	NA	NA	NA	NA	NA
23. Summary of main results		C	C	C	C	C	C
24. Effect of uncertainty		PC	PC	NR	PC	PC	PC
25. Effect of engagement with patients and others affected by the study		NA	NA	NA	NA	NA	NA
26. Study findings, limitations, generalizability, and current knowledge		PC	C	PC	C	C	C
27. Source of funding		NR	NR	NR	NR	NR	C
28. Conflicts of interest		NR	NR	NR	NR	NR	C
Score	The complete coincidence rate	38.1%	38.1%	28.6%	47.6%	50.0%	68.2%
	The total coincidence rate	76.2%	66.7%	52.4%	66.7%	72.7%	90.9%

Conducted (C): The report content adheres to the standard of a specific item on the CHEERS 2022 checklist; Partially conducted (PC): The report content is not comprehensive and partially adheres to the standard of a specific item on the CHEERS 2022 checklist; Not reported (NR): The report content does not comply with the standard of a specific item on the CHEERS 2022 checklist; Not applicable (NA): The content of the report does not relate to the standard of a specific item on the CHEERS 2022 checklist and the content does not apply to an item.

The assessment results of the CHEERS2022 scale of each included research showed that the complete coincidence rate was greater than 50%. The complete and total coincidence rates were employed to evaluate the quality of economic studies. The calculations were as follows:

The complete coincidence rate = number of conducted entries/(the total number of evaluation entries − number of un-applicable entries)*100%

The total coincidence rate = (number of conducted entries + number of partially conducted entries)/(the total number of evaluation entries – number of un-applicable entries)*100%

From the evaluation results, none of the included economics studies indicated the existence of a health economic analysis plan (100%). The majority of studies lacked information on relevant aspects, including the study perspective (83.33%), discount rate (83.33%), currency, price date, and conversion (66.67%), source of funding (83.33%), and conflicts of interest (83.33%). Additionally, a portion of the studies did not describe the methods employed for analyzing sources of uncertainty methods (33.33%), did not analyze the impact of uncertainty (16.67%), had no valuation of outcomes (33.33%), and failed in measurement and evaluation of resources and costs (16.67%).

### 3.4. Effectiveness of clinical treatment

The effectiveness indicators included in the study are detailed in Table 5.

**Table 5:** Efficiency and safety evaluation of included SR/meta-analysis.

Index	Study	Effect	95% CI	*P*	Note
The overall clinical efficacy rate	Tang, R 2017^[19]^	2.85	1.75, 4.63	*<*.001	
	Yu, F 2018^[20]^	1.22	1.17, 1.28	*<*.00001	Control: blank control, merger effect
		1.25	1.13, 1.38	*<*.0001	Control: blank control within 48 hours of cerebral infarction
		1.21	1.15, 1.27	*<*.00001	Control: blank control after 48 hours of cerebral infarction
		1.21	1.10, 1.32	*<*.0001	Control: Citicoline
		0.75	0.67, 0.84	*<*.00001	Control: Edaravone (prevailing side)
NIHSS score	Wang, Z 2017^[16]^	1.03	0.83, 1.28	.77	
	Bornstein, NM 2017^[17]^	0.60	0.56, 0.64	*<*.0001	
		0.54	0.49, 0.59	.10	z
		0.64	0.57, 0.72	.0001	Early changes of NIHSS in patients with moderate to severe stroke
	Guekht, A 2017^[18]^	0.59	0.53, 0.64	.0016	Baseline change in the NIHSS on the 14th day
		0.59	0.54, 0.64	.001	Baseline change in the NIHSS on the 21st day
	Tang, R 2017^[19]^	−1.77	−2.33, −1.21	*<*.001	
	Yu, F 2018^[20]^	−2.21	−3.56, −0.85	–	
MESSS score	Yu, F 2018^[20]^	−4.44	−6.55, −2.34	–	
ARAT score	Guekht, A 2017^[18]^	0.62	0.57, 0.68	*<*.0001	All randomized patients
		0.61	0.54, 0.68	.0015	ARAT baseline score > 0
BI score	Wang, Z 2017^[16]^	0.96	0.84, 1.08	.44	
	Zhang, D 2017^[15]^	6.80	−0.55, 14.16	.07	
	Tang, R 2017^[19]^	7.30	3.48, 11.13	*<*.001	
	Yu, F 2018^[20]^	4.34	3.15, 5.53	–	
mRS score	Bornstein, NM 2017^[17]^	0.61	0.52, 0.69	.01	
	Wang, Z 2017^[16]^	1.33	0.79, 2.24	.28	
	Zhang, D 2017^[15]^	1.32	0.88, 1.99	.18	Two-category data analysis
		−0.49	−1.21, 0.24	.19	Continuous data analysis
Whole blood viscosity	Yu, F 2018^[20]^	−0.66	−0.89, −0.43	*<*.00001	High shear rate
		−1.28	−1.86, −0.69	*<*.0001	Low shear rate
Fibrinogen content	Yu, F 2018^[20]^	−0.75	−1.19, −0.31	.0009	
Plasma viscosity	Yu, F 2018^[20]^	−0.27	−0.74, −0.20	.26	
Mortality rate	Zhang, D 2017^[15]^	0.82	0.55, 1.22	.33	
	Wang, Z 2017^[16]^	0.86	0.57, 1.31	.49	
	Bornstein, NM 2017^[17]^	0.81	0.50, 1.31	.49	
	Ziganshina, LE 2020^[21]^	0.90	0.61, 1.32	.58	
	Strilciuc, S 2021^[22]^	0.83	0.57, 1.23	.36	
	Tang, R 2017^[19]^	0.79	0.52, 1.19	.25	
Adverse reactions/adverse events	Zhang, D 2017^[15]^	0.98	0.90, 1.08	.75	
	Wang, Z 2017^[16]^	0.98	0.88, 1.09	.67	
	Bornstein, N. M 2017^[17]^	1.02	0.83, 1.26	.84	Fixed effect model
		0.99	0.70, 1.38	.94	Random effect model
	Ziganshina, L. E 2020^[21]^	0.97	0.85, 1.10	.62	
	Strilciuc, S 2021^[22]^	0.98	0.88, 1.09	.73	
	Tang R 2017^[19]^	1.04	0.85, 1.27	.72	
	Men P 2016^[28]^	1.37	0.95, 1.97	>.05	
Serious adverse reactions/serious adverse events	Zhang, D 2017^[15]^	1.18	0.85, 1.64	.31	
	Wang, Z 2017^[16]^	1.20	0.86, 1.66	.29	
	Bornstein, NM 2017^[17]^	1.08	0.73, 1.59	.70	
	Ziganshina, LE 2020^[21]^	1.15	0.81, 1.65	.44	
		2.15	1.01, 4.55	.047	Non-fatal serious adverse event
		0.90	0.59, 1.38	.63	Fatal, serious adverse event
	Strilciuc, S 2021^[22]^	0.99	0.74, 1.32	.95	
	Tang, R 2017^[19]^	0.01	−0.02, 0.04	.51	
	Men P 2016^[28]^	0.96	0.83, 1.11	>.05	
	Strilciuc, S 2021^[22]^	1.18	0.75, 1.86	.46	
Disability rate	Tang R 2017^[19]^	0.46	0.2, 1.03	.06	
Non-fatal loss	Ziganshina, L. E 2020^[21]^	0.97	0.45, 2.06	.93	

#### 3.4.1. Clinical effective rate.

Two Meta-analyses were included to examine the clinical efficacy rate of Cerebrolysin in the AIS. These researches indicated that combining Cerebrolysin with conventional treatment is significantly more effective than conventional therapy alone^[19,20]^ or a placebo combination^[19]^ in treating patients with AIS. A statistically significant difference in clinical efficacy rates was observed between the treatment and control groups (*P* < .05). The study by Yu et al,^[20]^ that administering Cerebrolysin to patients with cerebral infarction can enhance neurological function restoration and facilitate the successful recovery of daily life capabilities. However, subgroup analysis based on the timing of administration, whether preceding 48 hours post-cerebral infarction., revealed no significant difference in prognosis. In addition, in the group receiving conventional treatment in combination with other neuroprotective drugs, the therapeutic effect of Cerebrolysin was superior to citicoline diphosphate choline but inferior to Edaravone. The difference was statistically significant (*P* < .05).

#### 3.4.2. Neurological deficit assessment.

The higher the NIHSS and MESSS scales score, the more severe the central nervous system damage.^[33,34]^ Five meta-analyses in the treatment of AIS with Cerebrolysin were included. Four of these studies demonstrated that Cerebrolysin combined with the conventional treatment could reduce NIHSS^[17–20]^ and MESSS^[20]^ scores. The reduction in neurological deficit assessment scale scores showed significantly superior improvement compared to conventional therapy alone and a combined placebo treatment. These differences were statistically significant (*P* < .05). Two studies evaluated the early benefits of Cerebrolysin treatment based on changes in the NIHSS scores on the 14th^[18,20]^ and 21st days.^[18]^ These findings suggested combining Cerebrolysin with conventional treatment enhanced early neurological deficits in AIS patients. In the study by Bornstein et al,^[17]^ subgroup analysis based on earlier (day 21/30) NIHSS stroke severity was conducted. The results showed that early NIHSS changes in mild stroke patients treated with conventional treatment in combination with Cerebrolysin were similar to placebo, with no statistically significant differences(*P*>.05). Nonetheless, in patients with moderate to severe strokes, there was a statistically significant improvement in early NIHSS scores compared to placebo (*P* < .05). On the contrary, the study by Wang et al^[16]^ yielded different results, indicating no significant difference in NIHSS scores on the 90th day between the Cerebrolysin and placebo groups. However, subgroup analysis based on mild to moderate and severe symptoms for baseline stroke severity showed that Cerebrolysin demonstrated significant advantages in patients with severe AIS (NIHSS ≥ 8).

#### 3.4.3. Stroke-related motor outcome measure.

ARAT assesses upper extremity functional abilities through biomechanical analysis. It provides a score ranging from 0 to 57, with higher scores indicating better upper extremity function. A maximum score reflects the absence of upper limb dysfunction.^[23]^ The study by Guekht A et al^[18]^ assessed baseline change of ARAT on the 90th day in the subgroup of ARAT baseline score > 0 and all randomized patients, and the research results indicated that Cerebrolysin treatment showed the advantage of small to moderate, with a statistically significant difference(*P* < .05).

#### 3.4.4. Daily living ability assessment.

When assessing functional outcomes in stroke patients, the BI scores range from 0 to 100, with lower scores indicating more significant disability. The mRS consists of 7 levels, with higher levels showing more severe disability.^[24,34]^ Among the 5 studies included, the research conducted by Tang et al^[19]^ and Yu et al^[20]^ revealed that the BI score of Cerebrolysin in combination with conventional treatment was significantly higher than conventional therapy alone or in combination with a placebo treatment. These differences were statistically significant (*P* < .05). However, the findings from Wang et al^[16]^ and Zhang et al^[15]^ regarding the BI and mRS scores manifested that the recovery of activities of daily living of Cerebrolysin was similar to placebo, with no statistically significant differences(*P* > .05). The study by Bornstein et al,^[17]^ which conducted a long-term (at 90 days) analysis of mRS, also indicates that Cerebrolysin holds an advantage in improving disability for patients with moderate to severe strokes (NIHSS > 12).

#### 3.4.5. Hemorheology-related indicators.

The study by Yu et al^[20]^ indicated that Cerebrolysin combined with the conventional treatment significantly reduced both high and low shear rate blood viscosity and fibrinogen content compared to conventional therapy alone, with statistically significant differences(*P* < .05). However, there was no significant advantage in the Cerebrolysin group for plasma viscosity in patients with cerebral infarction.

### 3.5. Safety of clinical medication

Eight studies were included to assess the safety indicators of Cerebrolysin, comprising 7 meta-analyses and one pharmacoeconomic evaluation. The main findings of the studies showed no increase in mortality,^[15–17,19,21,22]^ the rate of AR^[19,28]^ or risk of AE,^[15–17,21,22]^ the rate of SAR,^[19,28]^ or the risk of SAE^[15–17,21,22]^ in the group of conventional treatment combined with Cerebrolysin when compared to conventional treatment alone or the combination of a placebo. These differences were not statistically significant (*P* > .05). The study by Yu et al^[20]^ reported AR when combining conventional treatment with Cerebrolysin. These reactions were all mild and self-resolving, with no reports of SAR. Commonly reported adverse effects included localized symptoms such as dizziness, headache, skin flushing, nausea, and systemic symptoms like fatigue and insomnia. In the research conducted by Ziganshina et al,^[21]^ a subgroup analysis was performed to evaluate SAE categorized as “fatal” and “non-fatal.” The results showed that the risk of fatal SAE after treatment with Cerebrolysin was similar to that of the placebo treatment, with no statistically significant difference between the 2 groups (*P* > .05). Nevertheless, the count of patients treated with Cerebrolysin for non-fatal SAE more than tripled. The subgroup analyses based on various dosing regimens for Cerebrolysin showed a significant increase in the risk of non-fatal SAE in the “30 mL for ten days” subgroup exclusively. The results of the study by Strilciuc et al^[22]^ indicated the following findings: compared to the placebo, the highest dose of Cerebrolysin (50 mL) had the lowest occurrence rate of SAE and non-fatal SAE, with a risk reduction of > 25%; the overall mortality rate showed a decreasing trend, with a risk reduction of 17%; Cerebrolysin demonstrated an advantage in reducing the risk of SAE in patients with moderate to severe stroke (NIHSS ≥ 8). A few literature studies reported that there were no differences in disability rate^[19]^ and non-fatal loss^[21]^ between conventional treatment combined with Cerebrolysin and combined with placebo. The safety indicators included in the study are detailed in Table 5.

### 3.6. Economy of therapeutic drugs

Six pharmacoeconomic evaluations of Cerebrolysin intervention were included, all adopted cost-effectiveness analysis. The study by Strilciuc et al^[30]^ demonstrated that, at a cost-effectiveness threshold of 50,000 euros, the addition of Cerebrolysin treatment showed favorable cost-effectiveness for moderate to severe AIS. The research findings from Men et al^[28]^ and He et al^[29]^ both support that, compared to conventional treatment alone, the incremental cost-effectiveness ratio (△C/△E) is lower with the combination of Cerebrolysin injection. The study by Lin et al^[25]^ suggested that in terms of significant efficacy and reasonable cost, Cerebrolysin was the preferred option compared with citicoline. Furthermore, relevant research has indicated that cost-effectiveness analysis results vary when Cerebrolysin treatments from different manufacturers are added to standard therapy. Lin et al’s study suggests that Lizhusaile is more cost-effective than Bi’aoxing.^[25]^ He et al’s research compared the efficacy of 3 brand names – Ningzexin, Shuruitai, and Qu’ao – and found that Ningzexin is the most economically favorable treatment for assisting in AIS.^[29]^ Cerebrolysin demonstrates superior cost-effectiveness compared to conventional drug therapy alone. However, related studies indicate its cost-effectiveness falls short of Edaravone injection and Gangliosides.^[26,27]^

## 4. Discussion

Currently, there is considerable debate regarding the utilization of neurotrophic medications in AIS treatment.^[8–10]^ Nevertheless, neuroprotective agents as supplementary therapy for AIS are widespread in clinical settings. Accordingly, this study is undertaken to conduct RHTA to evaluate neurotrophic drugs’ efficacy, safety, and cost-effectiveness. The objective is to furnish clinical physicians with pharmaceutical evidence to support the rational use of neurotrophic adjunctive medications.

Cerebrolysin treatment of AIS has a significant advantage in its efficacy among similar neuropeptide agents with neuroprotective effects. Zhang et al^[35]^ compared the efficacy of neuropeptide agents by using 2 drugs, Cortexin and Cerebrolysin, respectively, with saline control for treating right embolic middle cerebral artery occlusion (MCAO) rats. The study showed that only Cerebrolysin significantly improved the neurological prognosis after IS. Compared with other neuroprotective agents, Cerebrolysin still has some advantages in treating AIS. Mehta et al^[36]^ evaluated the efficacy of 4 neuroprotective drugs, including Citicoline, Edaravone, Cerebrolysin, and Minocycline, in patients with AIS in the middle cerebral artery (MCA) region. The results showed that the first 3 drugs reduced the mean NIHSS scores at day 11 and day 90 compared with placebo; the mean BI scores were increased, and the differences were statistically significant. However, the efficacy of Minocycline did not compare favorably with the neuroprotective agents mentioned above. According to Bogolepova et al^[37]^ rehabilitation of stroke requires a combination of treatments, including cognitive rehabilitation, motor rehabilitation and pharmacological correction, among others. The first choice for pharmacological correction is Cerebrolysin. In addition, Xue et al^[38]^ demonstrated that both DL-3-n-butylphthalide and Cerebrolysin could be used safely and contribute to improved neurological and behavioral outcomes in AIS patients, particularly in cases of moderate severity. However, Cerebrolysin was inferior to DL-3-n-butylphthalide in improving the short-term prognosis of AIS.

Most of the studies included in this study suggest that conventional treatment combined with Cerebrolysin improves the extent of neurological deficits and promotes the recovery of activities of daily living in patients with AIS, with a favorable safety profile; however, some of the studies concluded that the additional treatment with Cerebrolysin has no particular advantages and does not yet support the routine administration of Cerebrolysin to patients with AIS.

The results of this study showed that the ARAT score demonstrated the positive impact of Cerebrolysin on early motor function rehabilitation in AIS. NIHSS, BI, and mRS scores in some studies suggested that Cerebrolysin did not significantly affect the recovery of neurological function.^[15,16]^ However, subgroup analyses based on stroke severity have shown that Cerebrolysin has no significant therapeutic effect in mild stroke. It demonstrates an advantage in recovering and improving disabilities for patients with moderate to severe or severe AIS.^[16,17]^ Relevant research has also confirmed that the therapeutic effect of Cerebrolysin increases with stroke severity.^[7]^ In terms of safety, Cerebrolysin may increase the risk of non-fatal SAE.^[21]^ However, subgroup analyses based on the dose administered showed that the highest dose (50 mL) of Cerebrolysin had an advantage in reducing non-fatal SAE.^[22]^

Therefore, conclusions in the studies that do not support the clinical application of neurotrophic drugs^[15,16,21]^ should not be taken as dissenting opinions. These conclusions should be evaluated considering the specific research context. Additionally, it is essential to focus on recent, multi-center, large-sample, and high-quality clinical studies to enhance and update the research landscape. For instance, in the included studies, the conclusion of the study by Bornstein et al^[17]^ disagreed with the conclusions of Wang et al^[16]^ and Ziganshina et al^[39]^ (the conclusions of Ziganshina et al2020^[21]^ were essentially similar to the 2017 version^[39]^). The study by Bornstein et al presents conflicting evidence and demonstrates the beneficial impact of Cerebrolysin on the early improvement of overall neurological function in patients with AIS.

Clinical studies are still needed to compare Cerebrolysin with head-to-head comparisons in other neuroprotective agents, which needs more relevant literature. The latest report indicates that Cerebrolysin is currently in phase 3 clinical trials.^[40]^ Despite the encouraging results of the early trials of Cerebrolysin, more data from phase 3 randomized controlled trials are needed to support the clinical use of Cerebrolysin in treating AIS. In addition, more in-depth basic experiments on the mechanism of pharmacological effects are needed to clarify the efficacy and other potential roles of this drug for treating IS and to provide the best pharmacological treatment option for stroke patients.

## 5. Conclusion

In this study, we evaluated the efficacy, safety, and cost-effectiveness of Cerebrolysin for the treatment of AIS. The Meta-analyses/systematic evaluation results showed that Cerebrolysin was effective in treating AIS, significantly benefiting patients with moderate to severe IS. It could improve the degree of neurological deficit, promote the recovery of daily living ability, reduce blood viscosity and fibrinogen content, and improve the prognosis of patients. In terms of safety, Cerebrolysin exhibited a comparable safety profile to both the control and other positive groups, with a certain tolerance. In terms of economy, most studies suggest that Cerebrolysin offers an economic advantage. Nevertheless, it does not exhibit an economic advantage compared to certain neuroprotective drugs, such as Edaravone. Consequently, the use of Cerebrolysin should be approached comprehensively and judiciously, with the selection of the appropriate medication tailored to the patient’s specific medical condition and financial capacity.

This study employed a rapid assessment research methodology based on the RHTA process to collect literature, integrate and analyze data, and assess quality. In comparison to traditional HTA and SR, RHTA offers decision-makers timely technical information to facilitate decision-making while maintaining scientific rigor. However, it is essential to exercise caution when interpreting results, as rapid assessment methods have limitations in terms of inference due to database restrictions and data integration through descriptive analysis. Moreover, our study predominantly included literature in Chinese and English, influenced by language constraints, potentially introducing bias and limiting the generalizability of findings. Future research endeavors should overcome these challenges, providing more precise evidence for systematic evaluations of Cerebrolysin in treating AIS.

## Acknowledgments

We want to thank all the authors who participated in this study.

## Author contributions

**Conceptualization:** Xia Jiang.

**Investigation:** Yuqing Wei, Yeqian He.

**Supervision:** Xia Jiang.

**Validation:** Gonghao Zhang, Chunxia Yang.

**Writing – original draft:** Miaomiao Wan, Ke Yang.

**Writing – review & editing:** Miaomiao Wan, Ke Yang.

## Supplementary Material


