# Supplementary material for: Efficacy, safety, and cost-effectiveness analysis of Cerebrolysin in acute ischemic stroke: A rapid health technology assessment
Source: Medicine (Baltimore). 2024 Mar 29;103(13):e37593. doi: 10.1097/MD.0000000000037593 (PMC10977584; doi:10.1097/MD.0000000000037593)
Supplement: Supplementary file 1 [file medi-103-e37593-s001.docx]

| Table S1. Retrieval strategy in PubMed | |
| --- | --- |
| Steps | Retrieval strategy |
| #1 | "Stroke"[Mesh] |
| #2 | "cerebrovascular accident"[Title/Abstract] OR "CVA"[Title/Abstract] OR "Strokes"[Title/Abstract] OR "Apoplexy"[Title/Abstract] OR "Cerebrovascular Apoplexy"[Title/Abstract:~1] OR "Brain Vascular Accident"[Title/Abstract:~1] OR "Cerebrovascular Stroke"[Title/Abstract:~1] OR "Cerebral Stroke"[Title/Abstract:~1] OR "Acute Stroke"[Title/Abstract:~1] OR "Acute Cerebrovascular Accident"[Title/Abstract:~1] OR "Ischemic Stroke"[Title/Abstract:~1] OR "Ischaemic Stroke"[Title/Abstract:~1] OR "Cryptogenic Ischemic Stroke"[Title/Abstract:~1] OR "Cryptogenic Stroke"[Title/Abstract:~1] OR "Cryptogenic Embolism Stroke"[Title/Abstract:~1] OR "Wake up Stroke"[Title/Abstract:~1] OR "Acute Ischemic Stroke"[Title/Abstract:~1] OR "transient ischemic attack"[Title/Abstract] |
| #3 | "Protein Hydrolysates"[Mesh] |
| #4 | "Protein Hydrolysate"[Title/Abstract:~1] OR "cerebroprotein hydrolysate"[Title/Abstract] OR "cerebrolysin ebewe"[Title/Abstract] OR "cerebroly*"[Title/Abstract] OR "FPF1070" [Title/Abstract] |
| #5 | "Economics, Pharmaceutical"[Mesh] |
| #6 | "pharmaceutical economics"[Title/Abstract] OR "Pharmacoeconomics"[Title/Abstract] OR "Pharmacy Economic"[Title/Abstract:~1] OR "Cost Effectiveness"[Title/Abstract:~1] OR "Cost Effectiveness Ratio"[Title/Abstract:~1] OR "Cost Benefit Analysis"[Title/Abstract:~1] OR "Cost Benefit Analyses"[Title/Abstract:~1] OR "Cost Utility Analysis"[Title/Abstract:~1] OR "cost utility analyses"[Title/Abstract] OR "cost benefit"[Title/Abstract] OR "Costs and Benefits"[Title/Abstract:~1] OR "Cost and Benefit"[Title/Abstract:~1] OR "Marginal Analysis"[Title/Abstract:~1] OR "marginal analyses"[Title/Abstract] OR "Economic Evaluation"[Title/Abstract:~1] OR "cost" [Title/Abstract] |
| #7 | #1 OR #2 |
| #8 | #3 OR #4 |
| #9 | #5 OR #6 |
| #10 | #7 AND #8 AND #9 |
| #11 | #7 AND #8 AND #9 AND (meta-analysis[Filter]) |

This table is a search strategy for meta-analysis of Cerebrolysin in the treatment of Acute ischemic stroke in Pubmed.
